# Robotic versus Open Surgery in Locally Advanced Non-Small Cell Lung Cancer: Evaluation of Surgical and Oncological Outcomes

**DOI:** 10.3390/curroncol30100658

**Published:** 2023-10-12

**Authors:** Carmelina C. Zirafa, Gaetano Romano, Elisa Sicolo, Elena Bagalà, Beatrice Manfredini, Greta Alì, Andrea Castaldi, Riccardo Morganti, Federico Davini, Gabriella Fontanini, Franca Melfi

**Affiliations:** 1 Minimally Invasive and Robotic Thoracic Surgery, Surgical, Medical, Molecular, and Critical Care Pathology Department, University Hospital of Pisa, 56124 Pisa, Italy; gaetano.romano@ao-pisa.toscana.it (G.R.); elisa.sicolo92@gmail.com (E.S.); elenabagala98@gmail.com (E.B.); a.castaldi@studenti.unipi.it (A.C.); f.davini@ao-pisa.toscana.it (F.D.); franca.melfi@unipi.it (F.M.); 2 Division of Thoracic Surgery, Department of Medical and Surgical Sciences, University of Modena and Reggio Emilia, 41121 Modena, Italy; beatrice.manfredini91@gmail.com; 3 Pathological Anatomy, Surgical, Medical, Molecular, and Critical Care Pathology Department, University Hospital of Pisa, 56124 Pisa, Italy; greta.ali@gmail.com (G.A.); gabriella.fontanini@unipi.it (G.F.); 4 Section of Statistics, University Hospital of Pisa, 56124 Pisa, Italy; r.morganti@ao-pisa.toscana.it

**Keywords:** locally advanced non-small cell lung cancer, NSCLC, robotic surgery, minimally invasive surgery (MIS), surgical results, oncological outcomes

## Abstract

Locally advanced non-small cell lung cancer (NSCLC) consists of a heterogeneous group, with different pulmonary extension and lymph nodal involvement. Robotic surgery can play a key role in these tumours thanks to its technological features, although open surgery is still considered the gold-standard approach. Our study aims to evaluate the surgical and oncological outcomes of locally advanced NSCLC patients who underwent robotic surgery in a high-volume centre. Data from consecutive patients with locally advanced NSCLC who underwent robotic lobectomy were retrospectively analysed and compared with patients treated with open surgery. Clinical characteristics and surgical and oncological information were evaluated. From 2010 to 2020, 131 patients underwent anatomical lung resection for locally advanced NSCLC. A total of 61 patients were treated with robotic surgery (46.6%); the median hospitalization time was 5.9 days (range 2–27) and the postoperative complication rate was 18%. Open surgery was performed in 70 patients (53.4%); the median length of stay was 9 days (range 4–48) and the postoperative complication rate was 22.9%. The median follow-up time was 70 months. The 5-year overall survival was 34% in the robotic group and 31% in the thoracotomy group. Robotic surgery can be considered safe and feasible not only for early stages but also for the treatment of locally advanced NSCLC.

## 1. Introduction

Surgical treatment, in particular anatomical lung resection, is currently considered the gold-standard option for resectable non-small cell lung cancer (NSCLC). In the last decades, the introduction of minimally invasive surgery (MIS) has allowed surgeons to perform effective and safe procedures, avoiding the trauma related to the open approach. Robotic surgery is considered the evolution of the more established MIS and has been developed to overcome the technical limitations of video-thoracoscopy, maintaining the advantages due to its low invasiveness [1]. The high-definition three-dimensional vision, the greater manoeuvrability and the tremor filtration are some of the major advantages of robotic technology. About twenty years after the first robotic lobectomy, robotic thoracic surgery has become globally widespread, representing an excellent instrument for surgeons. Although the cumulative experience is currently evolving, to this day, the robotic approach is mainly used by surgeons to treat early-stage NSCLC, while open surgery remains the chosen approach for locally advanced stages. Many authors have reported their experience with robotic lung resections for early-stage NSCLC, showing good results in terms of feasibility and safety, with analogous long-term outcomes to the open approach [2,3,4]. However, studies on locally advanced stages are still limited.

This retrospective cohort study aims to evaluate the feasibility and safety of robotic surgery in performing major lung resections in the more advanced stages (stage IIIA, selected stage IIIB/IVA) of lung cancer and to analyse the operative and postoperative results and long-term oncological outcomes.

## 2. Materials and Methods

A retrospective analysis was conducted using a database on the surgical treatment of NSCLC, covering consecutive patients who underwent surgery between March 2010 and December 2020 at our hospital. The study was conducted in accordance with the Declaration of Helsinki and approved by the institutional ethics committee of Comitato Etico di Area Vasta Nord Ovest (CEAVNO).

Patients with a clinical diagnosis of stage IIIA and selected IIIB N2 NSCLC, according to the 8th edition American Joint Committee on Cancer staging system, were selected for this study. Moreover, patients with a single metastasis (IVA), already treated at the time of surgical lung resection, presenting local stage IIIA or IIIB N2, were also included in the study. Exclusion criteria were patients who underwent non-anatomical lung resection; underwent induction chemoimmunotherapy/immunotherapy; had a diagnosis of a carcinoid tumour; had poor pulmonary (FEV1 < 1l, predicted postoperative FEV1 and DLCO < 40%) or cardiac reserve (based on cardiac function evaluation); had an ASA (American Society of Anesthesiologists) score of 4; or had concurrent other malignant disease. The preoperative diagnosis was obtained by bronchoscopy, endobronchial ultrasound-transbronchial needle aspiration (EBUS-TBNA) or computed tomography (CT)-guided needle biopsy. Clinical staging was based on brain/chest/abdomen CT scan and fluorodeoxyglucose positron emission tomography (FDG-PET). In the case of enlarged or PET-positive lymph nodes or central tumours, invasive lymph nodal staging by EBUS or surgical biopsy was performed. 

### 2.1. Surgical Details

After institutional tumour board evaluation, informed consent was obtained from all subjects. All the patients underwent anatomical lung resection with systematic lymphadenectomy with either the robotic approach or thoracotomy. The selection of the surgical approach was at the discretion of the surgeon. During the surgical procedure, vascular and bronchial structures were individually isolated and resected. 

Robotic technique. The patients were positioned in a lateral decubitus position with the operating table tilted at the tip of the scapula. A standardized port mapping was applied using a four-arm “totally endoscopic” approach [5]. CO_2_ insufflation (5 mmHg) was applied to increase the thoracic space. The da Vinci Si platform was the robotic system used until 2014; from 2015 on, da Vinci Xi was routinely employed.

Open technique. The patients were placed in a lateral decubitus position with the operating table flexed at the level of the tip of the scapula. The surgical incision (thoracotomy) was usually performed at the 5th intercostal space. 

Data regarding the clinical characteristics of patients (age, sex, body mass index, comorbidities and smoking habits) were recorded. The surgical details collected were surgical approach (robotic surgery or thoracotomy), operative time, extension of lung resection, conversion to open surgery, histopathological staging, intraoperative and postoperative complications and length of hospital stay. The postoperative complications were evaluated according to the Clavien–Dindo classification [6]. During the follow-up time, the patients underwent chest CT, abdomen CT and/or abdomen ultrasound. According to medical opinion, total body PET-CT, bronchoscopy and brain magnetic resonance imaging or CT scan were performed when useful. 

We collected data about lung cancer relapses and mortality to analyse disease-free survival (DFS), local recurrence-free survival (LRFS), metastasis-free survival (MtsFS) and overall survival (OS). Overall survival is defined as the time from surgical procedure to death from any cause, while disease-free survival refers to the time from operation until the recurrence of disease (or death). Local recurrence-free survival and metastasis-free survival are defined as the time from surgery until the first loco-regional or systemic recurrence, respectively.

### 2.2. Data Analysis

Categorical data were described by absolute and relative (%) frequency; continuous data were expressed with mean and standard deviation (SD). To compare the surgical techniques (open and robotic) with various characteristics, a chi-squared test and t-test for independent samples (two-tailed) were applied, respectively.

Survival curves were calculated with the Kaplan–Meier method. The OS univariate analysis was performed by Cox regression and the effect of the surgery on the overall survival, local recurrence-free survival and metastasis-free survival was adjusted for the T parameter. Logistic regression was used to evaluate other outcomes. Significance was set at 0.05, and all analyses were carried out with SPSS v.28 technology (SPSS, Chicago, IL, USA).

## 3. Results

From March 2010 to December 2020, 131 consecutive patients with clinical local stage IIIA and IIIB N2 NSCLC underwent anatomic lung resection, 61 (46.6%) of them with the robotic approach and 70 (53.4%) with thoracotomy.

### 3.1. Clinical Characteristics of the Patients

In the robotic group, there were 38 males (62.3%) and 23 females (37.7%), with a median age of 67.3 years (range 32–80). A total of 51 (72.9%) males and 19 (27.1%) females underwent open surgery, with a median age of 69.4 years (range 48–82).

Current or former smokers constituted 88.5% of the patients treated with robotic surgery and 82.9% of patients treated with thoracotomy. The two groups resulted in similar median BMI, Charlson comorbidity index (CCI) score and American Society of Anesthesiologists (ASA) score distribution (Table 1).

### 3.2. Operative and Postoperative Results

Robotic surgery. Surgical lung resections performed with the robotic approach were lobectomy in 54 cases (88.5%), bilobectomy in 3 (4.9%), segmentectomy in 2 (3.3%) and pneumonectomy in 2 (3.3%). Neoadjuvant chemotherapy was administered in 12.5% of patients. The median operative time was 256 min (range 120–440), including the docking/undocking time. The conversion to open surgery occurred in eight (13.1%) cases, due to technical challenges (large lymph nodes adherent to hilar structures, diffuse adhesions) in seven cases and bleeding in one case. The average length of stay was 5.9 days (range 2–27). 

In the robotic group, postoperative complications were observed in 11 (18%) patients: 3 grade II and 8 grade III (6 IIIA, 2 IIIB), according to the Claven–Dindo complication classification. The postoperative complications consist of prolonged air leak in seven (11.5%) patients and postoperative bleeding/anaemia requiring blood transfusion in four (6.5%) patients. 

In the evaluation of the data, a difference in the postoperative outcomes was observed concerning the different generations of the robotic platform used (da Vinci SI from 2010 to 2014 vs. da Vinci Xi from 2015 to 2020). The median operative time appeared lower in the second period of the robotic experience, resulting in 275 min (range 180–440) in 2010–2014 and 245 (range 165–380) in 2015–2020. The median length of stay appeared longer in the first phase: 10 days (range 4–27) versus 5.4 days (range 3–13). The conversion rate was 19% in the first period and 11% in the second. The postoperative complications requiring treatment were lower in the second period, being reported in 25% of the cases in patients treated from 2010 to 2014 and in 9% of the cases from 2015 onwards. 

There were no intraoperative deaths. The postoperative mortality at 30 days was 0%. R0 resection was achieved in all patients. Data regarding surgical procedures performed and histopathological details are reported in Table 2. Adjuvant therapy was administered in 28 cases (45.9%), following tumour board evaluation.

Open surgery. Among the patients who underwent lung resection by thoracotomy, lobectomy was performed in 63 (90%) cases, pneumonectomy in 3 (4.3%), bilobectomy in 2 (2.8%) and segmentectomy in 2 (2.8%). Neoadjuvant chemotherapy was used to treat 18.4% of patients. The median operative time was 131 (range 60–230) minutes. After open surgery, the median length of hospital stay was 9 days (range 4–48), while the postoperative complication rate was 22.9%. In detail, grade II complications occurred in 10 patients and grade III complications (5 IIIA, 1 IIIB) in 6 patients. The most frequent postoperative complication was prolonged air leak, which was observed in eight (11.4%) patients. Moreover, postoperative bleeding/anaemia requiring blood transfusion was observed in six (8.6%) patients, bronchopleural fistula in one (1.4%) patient and atrial fibrillation in one (1.4%) patient.

There were no intraoperative deaths. The postoperative mortality at 30 days was 0%. R0 resection was achieved in all cases. Data regarding surgical procedures performed and histopathological features are reported in Table 2. Adjuvant therapy was administered in 27 (38.6%) cases, depending on the pathological stage and the clinical conditions of the patients.

The analysis of the confounding factors, in relation to the different surgical approaches, demonstrated that no statistically significant differences were observed for age (*p* = 0.134), sex (*p* = 0.196), smoking behaviours (*p* = 0.635), histology (*p* = 0.100), neoadjuvant chemotherapy (*p* = 0.419), adjuvant therapy (*p* = 0.341) or lymph nodal involvement (*p* = 0.804).

A different distribution of the T parameter was observed in the two groups, showing that T1–T2 tumours were mainly treated with robotic surgery (52.5% versus 47.5%), while T3-T4 lesions were predominantly managed with thoracotomy (59% versus 41%). Moreover, no significant differences were noted in the evaluation of the incidence of the most common complications, which were prolonged air leak (*p* = 0.704) and anaemia requiring blood transfusion (*p* = 0.645).

### 3.3. Oncologic Outcomes

**Figure 1:**
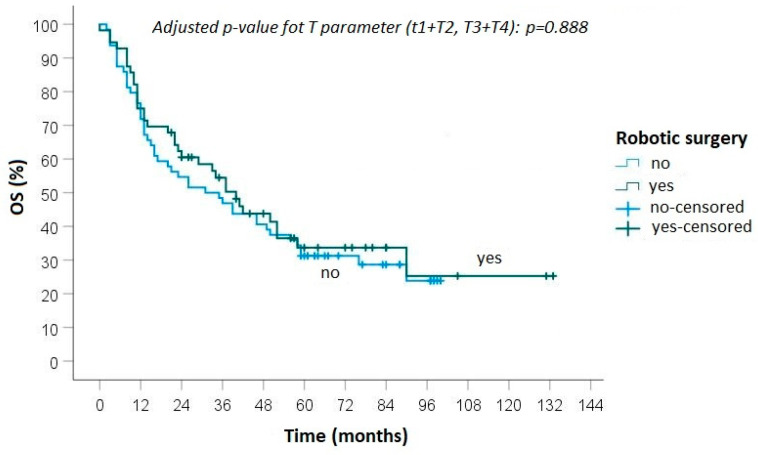
Overall survival between robotic and open surgery.

The median follow-up time was 70 months (range 21–133). The median DFS was 25.2 months after robotic lung resection, while it was 24.1 months after open surgery. The 5-year OS was 34.3% in the robotic group and 31.0% in the open surgery group (Figure 1). The univariate analysis of the predictive factors of overall survival is reported in Table 3.

**Figure 2:**
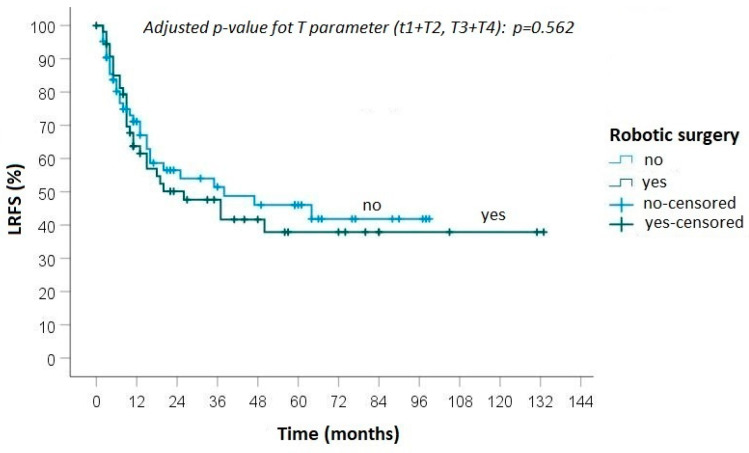
Kaplan–Meier curve for local recurrence-free survival between robotic and open surgery.

**Figure 3:**
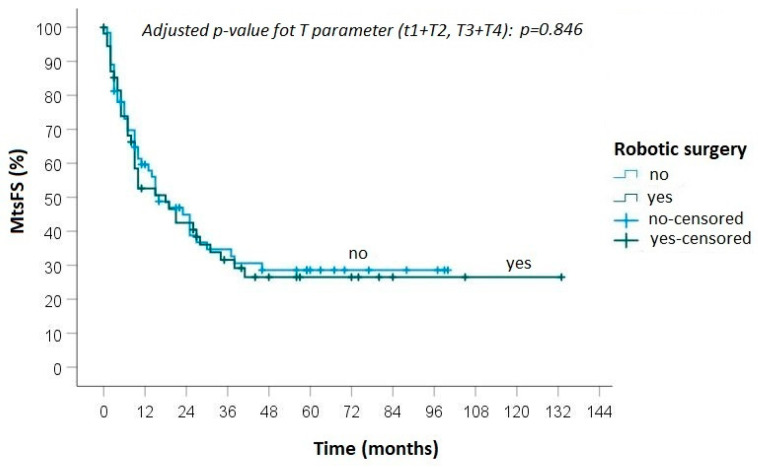
Kaplan-Meier curve for metastasis-free survival between robotic and open surgery.

During follow-up, progression of disease was detected in 40 (65.6%) patients treated with robotic surgery: local recurrence was diagnosed in 3 cases and distant metastasis in 11 cases, while in 26 cases both local and distant recurrences were observed (Figure 1 and Figure 2). In the group of patients who underwent open surgery, progression of disease was found in 48 (68.6%) cases: loco-regional recurrence was diagnosed in 7 patients, distant metastasis in 18 and an association of local and distant metastases in 23 (Figure 2 and Figure 3).

No statistically significant differences were found between the groups in terms of local recurrence (*p* = 0.50), distant metastasis (*p* = 0.88) and overall survival (0.82).

## 4. Discussion

At the beginning of the 1990s, the application of minimally invasive surgery in the thoracic field was described for the first time, and it quickly evolved by extending its use to more complex procedures, such as anatomic lung resection. Minimally invasive surgery presents several advantages over open surgery; it is in fact associated with reduced postoperative pain, shorter hospital stays and a lower complication rate [7,8,9]. 

The evolution of technology has led to the development of the robotic surgical system, characterized by high-definition 3D vision, tremor filtration and a wide range of articulation of the instruments. Thanks to its features, the robotic system is considered an advancement in the surgical field, exceeding the technical limits of conventional thoracoscopic surgery. Since 2001, when the first robotic lung lobectomy was reported, an increase in the application of the robotic approach in the treatment of NSCLC has been progressively documented [10]. Several authors have conducted studies on surgical and oncological results of robotic resections for lung cancer, reporting less postoperative pain, shorter hospitalizations and better aesthetic results in robotic surgery when compared to thoracotomy, with similar oncological outcomes [11,12,13]. 

Robotic surgery is thus considered a safe approach in the treatment of early-stage NSCLC, but data on its application in locally advanced-stage lung cancer are still lacking. Nevertheless, as suggested by the latest NCCN guidelines for lung cancer, the minimally invasive approach should be offered to all patients whenever possible [14]. Therefore, thanks to the advanced robotic technology and the growing expertise of surgeons, the indications of robotic surgery have also been extended to more advanced stages of NSCLC in recent years.

Locally advanced NSCLC is a heterogeneous disease cohort characterized by different sizes of tumours and lymph nodal involvement that constitutes about 30% of the non-small cell lung cancer patient population [15]. Given the characteristics of this group of tumours, multidisciplinary evaluation and treatment planning appear necessary to establish a suitable therapeutic strategy [16]. Although several innovations have been introduced in the field of cancer surgery, radiotherapy and medical treatment, the prognosis of locally advanced NSCLC patients has not changed over the past three decades, with a five-year OS of less than 10–40% [17].

Surgery is generally the first step in the treatment of stage III patients with N0/N1 disease, whereas the role of surgery is still an object of debate for patients with mediastinal nodal involvement [18]. The appropriate selection of patients to be treated with surgery appears fundamental to achieve local control, and the integration of surgery with a multimodality approach can have a beneficial impact on survival [19].

Surgical treatment must thus be focused on accuracy and radicality considering the variable survival rate associated with residual cancerous tissue. In particular, lymphadenectomy plays a decisive role in the prognosis, given that residual persistent N2 disease leads to a severe reduction in OS [20].

In recent years, technological advances have made minimally invasive surgery safe and effective, allowing for the extension of surgical indications to treat more complex cases. Locally advanced NSCLC patients can benefit from MIS thanks to the reduction in tissue trauma, a lower postoperative complication rate and faster recovery [21]. In the literature, some articles on the evaluation of patients with locally advanced-stage lung cancer treated by video-assisted thoracoscopic surgery are available. VATS was demonstrated to be safe with a low incidence of postoperative complications; furthermore, it appears to be equal to open surgery in terms of overall survival and disease-free survival [22,23]. 

Nevertheless, the quality of completeness of lymph node dissection achieved with VATS, which is a surrogate for the quality of surgery, is still being debated to date, as it is influenced by the surgeons’ expertise in minimally invasive surgical procedures [24]. 

As reported in several papers, robotic surgery allows the obtainment of a nodal upstaging similar to open surgery [25,26]. Thanks to its technical features, the robotic system can represent a precious instrument to approach locally advanced lung cancer using a minimally invasive technique. The surgeon, by performing procedures with the robotic system, is able to achieve more accurate dissection, with consequent proper completeness of resection, precise pathological stage of the disease and favourable postoperative outcomes, as our experience has also confirmed. Furthermore, the minimally invasive approach may play a relevant role in the optimization of therapy, reducing postoperative recovery and allowing the appropriate timing of adjuvant therapy [27].

Study on the application of robotic surgery in locally advanced lung cancer is still limited.

In 2016, Park showed similar hospital stay times, R0 resection rates, DFS and OS in a comparison between the outcomes of cII and cIIIA NSCLC patients, with 17 of them undergoing robotic surgery and 397 undergoing open surgery after induction therapy [28]. A low length of stay and a low complication rate were reported by Veronesi in a retrospective multicentre study including patients with clinically proven or occult N2 disease who underwent robotic-assisted surgery [29]. 

Locally advanced NSCLC represent a small group of patients among those eligible for surgery, and thus, while including a relatively limited number of patients, our analysis has described the larger monocentric experience published so far on the application of robotic surgery in locally advanced NSCLC compared with open surgery. We observed a reduction in the length of stay and postoperative complication rate in patients treated with the robotic approach when compared with open surgery, which is still considered the gold-standard approach for more advanced stages of lung tumours.

In our study, an evident difference in the length of stay and rate of conversion was observed between the first period (2010–2014), characterized by the use of the da Vinci Si surgical system, and the subsequent period (2015–2020), marked by the improved surgical expertise and the use of the newer da Vinci Xi surgical system. The data collected suggest that the technological advancement of the robotic platform in association with the evolution of surgical techniques may have impacted the improvement of postoperative outcomes. Furthermore, a reduction in the median operative time was noted in recent years, though it was still higher as compared to open surgery. The longer duration of the operation, mainly due to the docking/undocking of the system, represents a critical point of the robotic technique, influencing the total costs of the procedure. Nevertheless, in line with recent studies, the robotic approach is associated with a reduced number of conversions and a lower complication rate and length of hospital stay, allowing for reduced postoperative costs [30,31,32].

The positive postoperative data observed after robotic surgery are associated with favourable long-term results, and comparable outcomes were obtained with open surgery. In detail, a 5-year overall survival of 34% in the robotic group and 31% in the thoracotomy group was observed. The R0 rate and the 5-year OS reported in the robotic group, in line with the outcomes obtained after open surgery, suggest the potential to achieve a radical surgical procedure in more advanced stages.

On the contrary, lower overall 5-year mortality was observed after MIS lobectomy when compared to the open surgery lobectomy in a retrospective cohort analysis of 5741 cIIIA-N2 lung cancer patients from the national cancer database. The authors discussed the presumable role of potential confounders in these results, which was unverifiable due to specific missing data in the national database used for the analysis [33].

Instead, Li et al. confirmed that the robotic approach is safe and effective, with similar long-term outcomes when compared to VATS in stage IIB–IIIA NSCLC. In detail, the median DFS observed for the robotic and VATS groups was 31.1 and 33.8 months, respectively; the 3-year OS was 75.7% in the robotic group and 77.0% in the VATS group [34]. The safety and feasibility of robotic surgery in more advanced stages, with a high radicality rate, were confirmed by a recent study. In the evaluation of the outcomes of 95 IIB-IVA NSCLC patients, a 5-year recurrence-free survival of 36.7% was reported, which is in line with our result [35].

Some studies evidenced a small number of N2 patients who underwent induction therapy, and this trend was also observed in our study [36]. In our experience, patients with single mediastinal lymph nodal involvement, patients with necrotic/abscessualized tumours or patients temporarily unfit for chemotherapy have been addressed for upfront surgery. Furthermore, in this study, we decided to exclude patients treated with neoadjuvant immunotherapy, due to the peculiar and novel characteristics of this treatment and the favourable oncological outcomes reported by recent studies [37,38,39].

In our study, a positive trend was observed in surgical results after robotic surgery when compared to open surgery. In addition, no statistically significant differences were observed between the robotic and open groups regarding oncological outcomes. The results showed in this study are in agreement with the most consistent open surgery data, confirming that robotic surgery can allow accurate pathological staging and appropriate surgical radicality with faster postoperative recovery. This is useful in accelerating the administration of adjuvant treatment, which is fundamental to the multidisciplinary approach of locally advanced stages of disease. 

The main limitation of our study is its retrospective nature. Moreover, heterogeneity is an intrinsic characteristic of locally advanced NSCLC, which in itself represents a limitation of the analysis, although the two groups of patients were homogeneous in terms of clinical features. Additional data on a larger sample of patients will be necessary to confirm these results. Further studies are also necessary to merge surgery with novel systemic therapy and to optimize outcomes by tailoring the treatment.

## 5. Conclusions

The robotic system used by skilled surgeons also allows them to safely perform complex surgical procedures for the treatment of locally advanced NSCLC, which is associated with positive surgical and oncological outcomes. 

## Figures and Tables

**Table 1:** Clinical characteristics of the patients according to surgical approaches.

	Robotic Surgery (*n* = 61)	Open Surgery (*n* = 70)	*p*-Value
Sex			0.196
Male	38 (62%)	51 (73%)	
Female	23 (38%)	19 (27%)	
Age (years)	67.3	69.4	0.134
Smokers			0.635
Current	16 (26%)	17 (24%)	
Former	38 (62%)	41 (59%)	
FEV1 (litres)	2.51 (±0.77)	2.4 (±0.60)	0.613
BMI	26.6 (±4.67)	25.7 (±3.65)	0.210
ASA score			0.645
2	26 (43%)	26 (37%)	
3	35 (57%)	44 (63%)	
CCI score			0.532
0–4	46 (75%)	57 (81%)	
≥5	15 (25%)	13 (19%)	
T parameter			0.005
T1 + T2	32 (53.5)	20 (28.6%)	
T3 +T4	29 (47.5)	50 (71.4)	

FEV1: forced expiratory volume in one second; BMI: body mass index; ASA: American Society of Anesthesiologists; CCI: Charlson comorbidity index.

**Table 2:** Details of surgical procedures and histopathological diagnosis.

	Robotic Approach (*n* = 61)	Open Approach (*n* = 70)
Surgical procedure	2 segmentectomies	2 segmentectomies
	54 lobectomies	63 lobectomies
	11 RUL	22 RUL
	1 ML	4 ML
	18 RLL	10 RLL
	11 LUL	11 LUL
	13 LLL	16 LLL
	3 bilobectomies	2 bilobectomies
	2 pneumonectomies	3 pneumonectomies
Histology	Adenocarcinoma 46 (75.4%)	Adenocarcinoma 45 (64.2%)
	Squamous cell carcinoma 9 (14.7%)	Squamous cell carcinoma 21 (30%)
	Others 6 (9.8%)	Others 4 (5.8%)
Lymph nodes removed	18.9 (1–42)	19.4 (1–47)
N stations	5.1 (1–8)	5.1 (1–7)
N2 stations	3.3 (1–5)	3.2 (0–6)
pN	N0 5 (8.2%)	N0 4 (5.7%)
	N1 16 (26.2%)	N1 17 (24.3%)
	N2 40 (65.6%)	N2 49 (70%)
Pathological staging	IIIA 49 (80.3%)	IIIA 41 (58.6%) IIIB 29 (41.4%)
	IIIB 8 (13.1%)	
	IVA 4 (6.6%)	

RUL: right upper lobectomy; ML: middle lobectomy; RLL: right lower lobectomy; LUL: left upper lobectomy; LLL: left lower lobectomy.

**Table 3:** Univariate analysis of the OS predictive factors.

Factor	HR	95% CI–Lower	95% CI–Upper	*p*-Value
Robotic surgery: (0) no, (1) yes	0.891	0.574	1.385	0.609
Age	1.025	0.998	1.054	0.073
Gender: (0) M, (1) F	0.744	0.461	1.200	0.225
BMI	0.975	0.925	1.027	0.341
Smoker (0) no, (1) former, (2) yes	1.048	0.744	1.477	0.787
ASA score	1.570	1.004	2.455	0.048
Charlson comorbidity index	1.147	0.989	1.331	0.070
FEV1	0.945	0.691	1.292	0.723
Lobectomy	0.799	0.292	2.184	0.661
Adenocarcinoma	0.912	0.569	1.463	0.703
Squamous carcinoma: (0) no, (1) yes	1.200	0.717	2.008	0.488
N (0–2)	1.135	0.782	1.649	0.504
Stage (3–4)	0.807	0.294	2.215	0.677
T parameter: (0) T2 + T3, (1) T3 + T4	1.432	0.902	2.275	0.128

## Data Availability

The data presented in this study are available on request from the corresponding author. The data are not publicly available due to privacy reasons.
